# Addressing the tissue specificity of U5 snRNP spliceosomopathies

**DOI:** 10.3389/fcell.2025.1572188

**Published:** 2025-04-08

**Authors:** Rahmat Azhari Kemal, Raymond T. O’Keefe

**Affiliations:** ^1^ Division of Evolution, Infection and Genomics, Faculty of Biology, Medicine, and Health, School of Biological Sciences, University of Manchester, Manchester, United Kingdom; ^2^ Department of Medical Biology, Faculty of Medicine, Universitas Riau, Pekanbaru, Indonesia

**Keywords:** craniofacial malformation, pre-mRNA splicing, retinitis pigmentosa, spliceosomopathies, tissue specificity, U5 snRNP

## Abstract

Precursor mRNA (pre-mRNA) must undergo splicing to remove intron sequences and join exons. This splicing process is catalysed by an RNA/protein complex called the spliceosome. At the centre of the catalytic spliceosome is the U5 small nuclear ribonucleoprotein (snRNP). Pathogenic variants in U5 snRNP core proteins are associated with various diseases commonly known as spliceosomopathies. Variants in *TXNL4A* and *EFTUD2* manifest in craniofacial malformations while variants in *PRPF8* and *SNRNP200* manifest in retinitis pigmentosa. This perspective highlights research addressing how these specific manifestations come about as the spliceosome is required in all cells and at all developmental stages. Cell and animal models can replicate the human clinical specificity providing explanations for the specificity of the disorders. We propose that future research could benefit from models originating from patient-derived induced pluripotent stem cells (iPSCs) and isogenic controls to compare the coding and non-coding transcriptomic perturbations. Analysis of spliceosomal protein complexes and their interactome could also uncover novel insights on molecular pathogenesis. Finally, as studies highlight changes in metabolic processes, metabolomic studies could become a new venture in studying the consequences of U5 snRNP variants.

## 1 Introduction

Human genes, like other eukaryotes, contain translated exons and untranslated introns. To correctly produce the final messenger RNA and protein products, the precursor messenger RNA (pre-mRNA), as the initial product of transcription, must undergo splicing. The molecular mechanism of the splicing process has been reviewed elsewhere (Wan et al., 2020; Wilkinson et al., 2020). The RNA splicing process is catalysed by a complex RNA/protein machinery, the spliceosome. The spliceosome involves five small nuclear ribonucleoproteins (snRNPs) composed of small nuclear RNAs (snRNAs) and their respective associated proteins (Beusch and Madhani, 2024). In humans, the majority of introns (more than 99.5%) are spliced by the major spliceosome while the rest are catalysed by the minor spliceosome. The U5 snRNP is involved in the catalytic process of both spliceosomes (Akinyi and Frilander, 2021).

The U5 snRNP consists of the U5 snRNA, seven Sm proteins, and eight core proteins (Wood et al., 2021). The core U5 snRNP is assembled in the cytoplasm from an Sm protein ring and U5 snRNA and then imported to the nucleus. In nuclear Cajal bodies, the core U5 snRNP interacts with an RNA-free heterotetrameric complex consisting PRPF8, SNRNP200, EFTUD2, and SNRNP40. Afterwards, PRPF6, DDX23, CD2BP2, and TXNL4A join the complex. The final U5 snRNP then interacts with the U4/U6 di-snRNP to form U4/U6.U5 tri-snRNP (Klimešová et al., 2021; Riabov Bassat et al., 2024). In the central region of the tri-snRNP, PRPF8 serves as scaffold. It interacts and positions the U5 snRNA with exons. PRPF8 also interacts with EFTUD2 and regulates SNRNP200 ATPase activity (Kastner et al., 2019). TXNL4A is also located centrally in the tri-snRNP. For the formation of the catalytic spliceosome, TXNL4A must depart the spliceosome (Schreib et al., 2018).

Pathogenic variants affecting spliceosome components manifest in a wide range of diseases or disorders commonly known as spliceosomopathies (Love et al., 2023). Variants in *TXNL4A* and *EFTUD2* manifest in similar craniofacial malformations while variants in *PRPF8*, *SNRNP200*, and *PRPF6* result in retinal degeneration. These genetic disorders have been previously reviewed elsewhere (Beauchamp et al., 2020; Griffin and Saint-Jeannet, 2020; Maxwell et al., 2021; Wood et al., 2021). In this perspective, we would like to highlight research that has been conducted to address the specific disease manifestations as well as raise insights for future research. Due to space constraints, this perspective will not focus on *PRPF6*. For consistency, in this article, we use TXNL4A, EFTUD2, PRPF8, and SNRNP200 to refer to the human and animal proteins and Dib1, Snu114, Prp8, and Brr2 to its respective orthologue in yeast.

## 2 U5 snRNP components with craniofacial manifestation

### 2.1 TXNL4A

TXNL4A, orthologous to yeast Dib1, is one of the core proteins of the U5 snRNP (Liu et al., 2006). It stabilises U4/U6.U5 tri-snRNP complex, preventing premature spliceosome activation (Schreib et al., 2018). Variants in this gene cause Burn-McKeown syndrome (BMKS). The syndrome is a rare autosomal-recessive disorder with choanal atresia, craniofacial anomalies including lower eyelid coloboma and cleft lip, and other extra-craniofacial manifestations. Almost all patients have normal intellectual development with only one reported having intellectual disability (Wieczorek et al., 2014; Wood et al., 2022). Clinical manifestations arise from compound heterozygosity of a loss-of-function allele and either type 1 or type 2 promoter deletion or homozygosity of either type of promoter deletion (Wieczorek et al., 2014; Goos et al., 2017; Wood et al., 2022). Promoter deletion reduces TXNL4A levels shown by luciferase assays (Wieczorek et al., 2014; Wood et al., 2022).

Using frog *Xenopus*, Park et al. (2022) showed spatiotemporal specificity of *Txnl4a* during embryo development. While *Txnl4a* is expressed at all developmental stages, the gene is enriched at the anterior neural plate and the neural-crest-forming regions during the neurula stage. Knockdown of *Txnl4a* decreases the expression of several neural crest genes and negatively affects neural crest formation and survival, thus impacting craniofacial development (Park et al., 2022). As shown in mouse embryonic stem cells, the molecular pathogenesis involves mis-splicing of *Mdm2*, upregulation of p53, and downregulation of several glycolytic genes (Varineau and Calo, 2024). Utilising BMKS patient-derived induced pluripotent stem cells (iPSCs), Wood et al. (2020) found that patient cells have delayed proliferation and differentiation into neural crest cells (NCCs). The NCCs had delayed epithelial-to-mesenchymal transition which might be caused by downregulated WNT signalling via mis-splicing of *TCF7L2*. Both *in vitro* and animal model data indicate that neural crest developmental defect causes the craniofacial malformations in BMKS patients.

### 2.2 EFTUD2

EFTUD2 and its yeast orthologue Snu114 are GTP-bound proteins in the core of the U5 snRNP providing a scaffold for spliceosome complex assembly and disassembly (Jia et al., 2020). Variants that inactivate one allele causing haploinsufficiency in *EFTUD2* result in Mandibulofacial dysostosis Guion-Almeida type (MFDGA). Patients are characterized by malar and mandibular hypoplasia as the core craniofacial malformations as well as microcephaly, external ear malformations, and intellectual disability (Abell et al., 2021). The clinical phenotype hints at cell-type specific effects of *EFTUD2* variants. To study the molecular mechanism of *EFTUD2* variant, Wood et al. (2019) introduced a knockdown mutation in HEK293 human cell line. The knockdown caused changes not only in the expression level but also in the mis-splicing of genes associated with biological processes involved in craniofacial development. In a zebrafish model (Lei et al., 2017), apoptosis occurs in the head and spinal cord’s neuronal progenitors of the *Eftud2* mutant leading to smaller heads and abnormal brain development. Non-neuronal organ patterning and development had little or no changes. In a mouse model, similar tissue specificity of *Eftud2* expression was also observed. *Eftud2* was expressed in precursors of the brain, face, and head which are the affected organs of MFDGA patients. However, there was a lack of craniofacial malformation and p53 activation in the heterozygous mouse model. Additionally, homozygous mutants were lethal (Beauchamp et al., 2019). To address the lethality issue, neural crest cell-specific *Eftud2* homozygous mutants were developed using a Wnt1-Cre transgene (Beauchamp et al., 2021). The resulting mutants developed brain and craniofacial malformation, replicating the human phenotype.

*EFTUD2* molecular pathogenesis in neural crest cells is proposed to be mediated by p53 upregulation. In HEK293 cells, p53 was identified as a top upstream regulator of genes with increased exon skipping caused by *EFTUD2* knockdown (Wood et al., 2019). The p53 signalling pathway is also activated in neural cell progenitors by aberrant splicing in *Eftud2*-mutant zebrafish (Lei et al., 2017). The involvement of p53 was also supported by the improvement of craniofacial development by a p53 inhibitor in the mouse model (Beauchamp et al., 2021). Specifically, p53 pathway upregulation seems to be induced by exon 3 skipping in *Mdm2* (Beauchamp et al., 2021; Varineau and Calo, 2024). However, craniofacial malformations from *Eftud2* insufficiency can also be triggered by p53-independent pathways (Beauchamp et al., 2022). As *EFTUD2* knockdown elicits endoplasmic reticulum stress and unfolded protein responses in a human cell line (Wood et al., 2019), these pathways might contribute to the abnormal cellular processes leading to apoptosis of neural crest cells. While the cell line and animal models have provided additional insights on MFDGA pathogenesis, patient-derived iPSCs should be generated and differentiated into neural crest cells and other relevant cell types to better understand the molecular mechanisms of EFTUD2 variants.

## 3 U5 snRNP components with retinal manifestation

### 3.1 PRPF8

PRPF8, the human orthologue of yeast Prp8, occupies the catalytic centre of the spliceosome (Grainger and Beggs, 2005). Its N-terminal portion interacts with EFTUD2 (Jia et al., 2020) while its C-terminal Jab1/MPN domain regulates SNRNP200 ATPase activity (Mayerle and Guthrie, 2016). Variants in *PRPF8* cause retinitis pigmentosa type 13 (RP-13). This progressive retinal degeneration is inherited in an autosomal dominant manner (McKie et al., 2001). Specific manifestation of *PRPF8* variants in the retina might result from the increased splicing activity in retina. Compared to other cell types, retina highly expresses snRNAs and spliced transcripts of housekeeping/constitutive mRNAs (Tanackovic et al., 2011). The worm *C. elegans* model of *PRPF8* knockdown also revealed that defect phenotype occurs in a cell-type with high transcriptional activity (Rubio-Pena et al., 2015). Using fibroblasts from RP patients, Atkinson et al. (2024) generated iPSCs and used CRISPR-Cas9 correcting the variant in *PRPF8* to generate isogenic normal controls. Both iPSCs were differentiated into retinal pigment epithelium (RPE), retinal organoids, as well as kidney organoids. The study supported the tissue/organ-specific defects in RP where impaired alternative splice site selection and enhanced usage of cryptic/aberrant splice sites occur in RPE and retinal organoids compared to kidney organoids. The specificity might stem from special nuclear clusters of transcription and splicing machineries in human photoreceptor cells. RPE and retinal organoids derived from RP patient’s iPSCs had dispersed nuclear speckles, while kidney organoids from the same iPSCs had nuclear speckles structurally similar to controls (Atkinson et al., 2024). While this subcellular organisation facilitates higher efficiency in human photoreceptor cells, this organisation introduces a vulnerability to pathogenesis observed in RP.

*Drosophila* models expressing variant Prpf8 had increased apoptosis in developing eye primordium leading to smaller adult eyes. Interestingly, this apoptosis was only observed when the variant proteins were expressed early in the eye primordium but not when expressed late in the differentiated photoreceptors (Stanković et al., 2020). In line with the fly model, mouse models with heterozygous and homozygous *Prpf8* variants also had no photoreceptor degeneration. However, the retinal pigment epithelium (RPE) had degenerative ultrastructural changes that were more severe in the homozygous model (Graziotto et al., 2011). Using primary RPE culture from the homozygous variant model, Farkas et al. (2014) showed functional disturbance of adhesion and phagocytosis in the variant RPE’s photoreceptor outer segments. The animal models show that the RP manifestation is not only organ-specific, but also cell-type specific.

### 3.2 SNRNP200

SNRNP200, and its yeast counterpart Brr2, are helicases crucial for the snRNA unwinding process during spliceosome activation (Ledoux and Guthrie, 2016). Variants in *SNRNP200* cause retinitis pigmentosa type 33 (RP-33). This inherited retinal degeneration seems to have a dual inheritance pattern. Initially identified as autosomal dominant, there have been several reports indicating autosomal recessive inheritance pattern (Holtes et al., 2025). While the variants specifically affect the retina, *SNRNP200* is ubiquitously expressed in human, mice, and zebrafish (Zhao et al., 2009; Zhang T. et al., 2021). Both *SNRNP200* knockdown and *SNRNP200* variant expression resulted in systemic deformities in zebrafish embryos. The zebrafish model developed normal ocular size and cone photoreceptor morphology, but had significantly decreased rhodopsin (Liu et al., 2015; Zhang T. et al., 2021). Interestingly, while knockdown increased larvae deformities with moderate death, variant protein significantly increased larval death (Zhang T. et al., 2021). This observation supports the dominant-negative pattern of *SNRNP200*, as observed in humans.

As with other spliceosomal defects, the molecular pathogenesis might involve disturbance in cell cycle/growth. Expression of variant SNRNP200 in HEK293 cells resulted in observable cell dysmorphology (Ehsani et al., 2013). Cell cycle is also affected, with increased cell populations in G2/M phase compared to the control. On the other hand, knocking down *SNRNP200* resulted in decreased G1 phase and increased S phase but unchanged G2/M phase. This observation hints at a different mechanism between dominant-negative variant and knockdown. One putative mechanism is through more marked reduction of alpha-tubulin expression in cell lines expressing variant protein (Ehsani et al., 2013). Patient-derived iPSCs could be used to further study the *SNRNP200* variants mechanism in human cells and tissues (Zhang D. et al., 2021).

## 4 Discussion

Even though splicing is a ubiquitous process, different variants in the pre-mRNA splicing machineries result in different pathologies. Here we discuss two contrasting phenotypes from variants in U5 snRNP proteins. *TXNL4A* and *EFTUD2* manifest in craniofacial deformation which might arise from similar defects in cranial neural crest cell formation (Park et al., 2022). On the other hand, *PRPF8* and *SNRNP200* have an even more specific phenotype in the form of retinal dysfunction. Even within the same type of defect, the phenotypes are still distinct. BMKS is distinguishable from MFDGA based on microcephaly and developmental delay in MFDGA. Distinctive facial phenotypes such as lower eyelid coloboma in BMKS and malar hypoplasia in MFDGA also help to distinguish these craniofacial disorders (Lüdecke and Wieczorek, 2016; Abell et al., 2021). Differences between BMKS and MFDGA clinical phenotypes might be explained by unique gene expression changes (Park et al., 2022). Interestingly, *TXNL4A* and *EFTUD2* knockdowns shared common molecular mechanism. The splicing perturbation in the *Mdm2* gene leads to p53 upregulation followed by glycolytic transcript downregulation (Varineau and Calo, 2024). In contrast, different variants of the same gene might result in different transcriptional changes. In *Drosophila*, different *Prpf8* point-mutations had dissimilar differentially expressed genes with only limited overlap (Stanković et al., 2020). As more patients are identified and analysed, we might see distinct manifestations that might confront the cell specificity hypothesis. For example, a study reported retinal dystrophy in an MFDGA patient with a novel EFTUD2 variant, where the condition does not typically involve eye anomalies (Deml et al., 2015).

**FIGURE 1:**
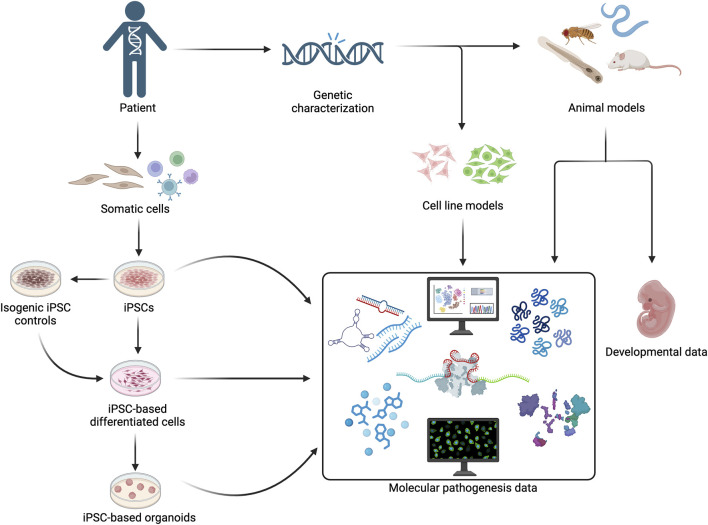
Patient-derived cells, human cell lines, and animal models can be utilised to obtain molecular pathogenesis and developmental manifestation addressing the tissue-specificity of U5 snRNP protein gene variants.

The variants in U5 snRNP components usually manifest in cell types and developmental stages that are relatively inaccessible in humans. Therefore, *in vitro* and animal models have been utilised to obtain insights on the effects of U5 snRNP component variants (Figure 1). Variants of interest can be introduced into established human cell lines. For example, Wood et al. (2019) utilised CRISPR-Cas modification to introduce *EFTUD2* haploinsufficiency in human embryonic kidney HEK293 cells. Moreover, patient-derived cells can be induced for pluripotency and differentiated into target cells, even organoids. Several studies have compared patient-derived iPSCs with healthy control-derived iPSCs, but this approach must account for different genetic background between patient and control cells. To address this issue, Atkinson et al. (2024) developed an isogenic normal control where the disease-causing variant is corrected by CRISPR-Cas9.

Most spliceosomopathies are studied *in vitro* by differentiating iPSC into the (putative) affected cell types. While this approach seems the most sensible approach, it has not fully addressed the specificity question. If variants in *PRPF8* manifest in retina as a result of its high splicing activity, why do variants in *TXNL4A* or *EFTUD2* not manifest in the retina as well? Atkinson et al. (2024) has tried to address the specificity by analysing *PRPF8* variants in unaffected tissue/organs. They suggested that different subcellular organization, such as of nuclear speckles, in different cell types could serve as the basis of cell specificity. However, future research could compare the manifestation of *TXNL4A* and *EFTUD2* variants in retinal cell/organ or *PRPF8* and *SNRNP200* variants in neural crest cell to gain more insights on the mechanisms. Additionally, since spliceosomopathies could affect several organs/systems, analysing the manifestation in more cell types could uncover the mechanism(s) behind the specificity. For example, osteoblast and chondrocyte cells could be utilized to study skeletal dysplasia in MFDGA (Wu et al., 2019). While RP does not usually have skeletal manifestation, *SNRNP200* knockdown inhibits osteo-/dentinogenic differentiation of stem cells from the apical papilla (Su et al., 2020). These studies show the benefit of expanding the cell type landscape in increasing our understanding on spliceosomopathies.

Spliceosomopathies manifest early in development. Since it is controversial to study the disease in developing human embryos, animal models have provided a way to study the effect of spliceosome gene variants in early development. Lower animal models such as *C. elegans* and *Drosophila* as well as higher animal models such as zebrafish, frog, and mice have been developed. A common challenge is the lethality of knockdowns or homozygous mutants. Several methods have been utilized to overcome this phenomenon such as using Wnt1-Cre transgene to develop cell-type specific mutants (Beauchamp et al., 2021) or utilising different promoter systems to express the target protein at different developmental stages (Stanković et al., 2020). Using those methods, animal models could be designed to observe the variant effects not only on specific cell types but also at specific developmental stages.

Once the disease models have been developed, various molecular analysis could be conducted (Figure 1). Transcriptomic and proteomic changes are measured in various studies with spliceosome dysregulation. While comparison between variant and healthy controls has been the norm, one might also add another layer of analysis such as different transcriptional and proteomic changes resulting from different variants of the same gene. For example, in *Drosophila*, the expression of more toxic *PRPF8* variants had only limited overlapping differentially expressed genes compared to a variant associated with mild or no human symptoms. Apoptosis and adult eye defect were induced by the more toxic variants but not by a milder one (Stanković et al., 2020). The information might explain the manifestation variability between various U5 snRNP variants. Comparing the molecular pathogenesis of different *SNRNP200* variants might answer the question of its dual inheritance pattern (Holtes et al., 2025). Another parameter of interest is the non-coding RNAs. It has been shown with PRPF8 that variant protein causes misexpression of circRNAs and degeneration of cerebellar granule cells in a mouse model (Krausová et al., 2023). The effect of other U5 snRNP variants on non-coding RNA should also be studied. SNRNP200 plays a role in plant miRNA biogenesis (Li et al., 2024), therefore variants affecting SNRNP200 activity might result in changes of miRNA expression. As mutating Dicer protein responsible for miRNA biogenesis results in neural crest cells defects and craniofacial anomalies (Huang et al., 2010), understanding the role of *TXNL4A* and *EFTUD2* variants on miRNA biogenesis in neural crest cells could open a new molecular mechanism in craniofacial malformations.

U5 snRNP variants affect spliceosome assembly or catalytic capacities. As shown in yeast, both *Dib1* and *Snu114* variants reduce the U5 and/or U4/U6.U5 tri-snRNP spliceosome complex assembly (Brenner and Guthrie, 2006; Wieczorek et al., 2014). The molecular mechanism behind this effect could be the decrease of functional proteins caused by loss of function, reduced expression from mutated promoter, or mis-splicing of the gene transcript (Wieczorek et al., 2014; Thomas et al., 2020; Wood et al., 2022). Meanwhile, variant PRPF8 and SNRNP200 proteins are well-incorporated into the U4.U6/U5 tri-snRNP complex (Tanackovic et al., 2011; Cvačková et al., 2014; Atkinson et al., 2024). However, the variants cause functional defects. *PRPF8* variants cause defects in the second catalytic step of splicing via deregulation of SNRNP200 helicase activity (Mozaffari-Jovin et al., 2013; Mozaffari-Jovin et al., 2014; Mayerle and Guthrie, 2016). *SNRNP200* variants with reduced ATPase activity had lower RNA duplex unwinding activity, which is crucial to unwind the U4/U6 duplex (Ledoux and Guthrie, 2016). In yeast, defective Brr2 unwinding activity inhibits the formation of catalytic spliceosomes (Zhao et al., 2009). Variants in the Brr2 RNA-binding pocket also seems to shorten the Brr2 interaction time with pre-mRNA (Cvačková et al., 2014).

Transcriptomic and proteomic analysis also hint at the effects of variants in an individual U5 snRNP to different snRNP components. *EFTUD2* knockdown decreases *TXNL4A* expression in HEK293 cells (Wood et al., 2019). *SNRNP200* knockdown in zebrafish upregulates several spliceosome components, such as *PRPF3*, *PRPF6*, *PRPF8*, and *PRPF31* (Liu et al., 2015). Since the spliceosome operates as a complex, a complexome type of analysis could be utilised to study the effect of variants on the complex dynamics (Wittig and Malacarne, 2021). Interactome analysis could also enrich the mechanistic insight behind variant U5 snRNPs pathogenesis. For example, a study showed that human cohesin protein interacts with EFTUD2 and SNRNP200, among several components of U4/U6.U5 tri-snRNP, which might explain cell cycle defect resulting from U5 snRNP variants (Kim et al., 2019). Since U5 snRNP is assembled in both the cytoplasm and nucleus (Matera and Wang, 2014), analysis on subcellular fractions should also be conducted. Study from another protein complex, the C-terminal to LisH (CTLH) E3 ligase complex, showed distinct assembly and interactomes in the cytoplasm and nucleus (Onea et al., 2022). Recent advances in spatial transcriptomics and spatial proteomics could also become useful tools to understand the contrasting craniofacial and retinal manifestations (Montgomery et al., 2022; Nalbach et al., 2023).

Finally, it has been shown that splicing perturbation caused by splicing factor knockdowns results in glycolytic transcript downregulation leading to cellular metabolic changes (Varineau and Calo, 2024). As glycolysis is important for neural crest migration (Bhattacharya et al., 2020) and survival of rod cells (Petit et al., 2018), its downregulation could also explain the molecular mechanism of U5 snRNP variants in the respective affected cells. Therefore, metabolomic studies could provide a novel point of view on the pathogenesis in spliceosomopathies.

The strategies discussed in this perspective are not only applicable for the four snRNP components described. The iPSC-derived RPE cells have been utilised to study the molecular pathogenesis of *PRPF6* in retinitis pigmentosa 60 (Liang et al., 2022). For other less characterised U5 snRNP components, studies could be initiated by collecting patient cohorts through GeneMatcher (Sobreira et al., 2015). This approach has identified an association of *DDX23* variants to a neurodevelopmental disorder (Burns et al., 2021). In mice, *CD2BP2* and *SNRNP40* knockout have been associated with podocyte and immune cell function, respectively (Albert et al., 2015; Zhang et al., 2019). Future studies could utilize GeneMatcher to identify and characterize patients with *CD2BP2* or *SNRNP40* variants. Once human association is found, further dissection of molecular and developmental pathogenesis could be investigated utilizing approaches outlined in this perspective.

## Data Availability

The original contributions presented in the study are included in the article/supplementary material, further inquiries can be directed to the corresponding author.
